# Supplementary material for: Physician preparedness for resource allocation decisions under pandemic conditions: A cross-sectional survey of Canadian physicians, April 2020
Source: PLoS One. 2020 Oct 22;15(10):e0238842. doi: 10.1371/journal.pone.0238842 (PMC7580904; doi:10.1371/journal.pone.0238842)
Supplement: S1 Appendix — (DOCX) [file pone.0238842.s001.docx]

**Preparing for Resource Rationing under Pandemic Conditions**

Start of Block: Default Question Block

Preparing for Resource Rationing under Pandemic Conditions   You are being invited to participate in a research study on resource allocation under pandemic conditions. You were selected to participate in this study because you are a staff physician in the Ottawa region.   Taking part in this study is voluntary.  Your participation or lack thereof will not affect your employment.
 You will be asked to describe your feelings surrounding resource allocation, your thoughts on policies about resource allocation, and your methods for determining who would get a limited resource under the pandemic conditions of COVID-19. You may find some of these questions upsetting, but you are free to stop at any time.   It will take you approximately 10 minutes to complete the survey.    You may not directly benefit from this research; however, we believe this research is crucial to understanding the decision-making of hospital physicians facing resource scarcity, which can in turn be used to design policy that reflects the perspectives of front-line staff.   We are committed to protecting your confidentiality. Your responses will be reported only in aggregate form, and we are not collecting names or other identifying information.   By beginning this survey, you will have been deemed to have given consent for your participation. If, at the end of the survey, you decide that you wish to withdraw consent, you can do so by indicating it in the final comment box.
 This study is being led by Dr. Michel Shamy from the University of Ottawa/The Ottawa Hospital/Ottawa Hospital Research Institute.  This project has received REB approval and is funded by a Department of Medicine SPARC grant. No industry funding was sought or received.

 If you have any questions, please contact me at 613-761-4709 or mshamy@toh.ca
If you have questions about your rights as a participant or about ethical issues related to this study and would like to speak to someone not involved in this study, please contact The Ottawa Health Science Network Research Ethics Board, Chairperson at 613-798-5555 extension 16719.  

 Thank you for your consideration and stay safe,
 Dr. Michel Shamy MD MA FRCPC

| Page Break |  |
| --- | --- |

1A Imagine that you have two patients who require a ventilator but only one ventilator is available. How prepared do you feel to determine who will receive the ventilator?

- Not at all prepared (1)
- A little prepared (2)
- Somewhat prepared (3)
- Very prepared (4)

| Page Break |  |
| --- | --- |

| 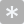 |
| --- |

1B Imagine that you will need to make a decision involving allocating a ventilator or ICU bed in the near future to one of two patients. In ONE WORD, please describe how you feel now, as you think about that decision.

________________________________________________________________

| Page Break |  |
| --- | --- |

| 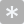 |
| --- |

1C Imagine that you have recently made a decision in which a patient was assigned an ICU bed and the other was palliated. In ONE WORD, please describe how you expect to feel in this situation:

________________________________________________________________

| Page Break |  |
| --- | --- |

2A To what extent do you agree or disagree with this statement: There should be hospital policies that determine who gets a ventilator, critical care bed or other limited resource in a time of pandemic.

- Strongly disagree (1)
- Somewhat disagree (2)
- Neither agree nor disagree (3)
- Somewhat agree (4)
- Strongly agree (5)

| Page Break |  |
| --- | --- |

2B As a reminder, your answers will be kept strictly confidential. If the hospital implemented a policy that **mostly aligned** with your beliefs on allocation of ventilators, critical care beds or other limited resources, would you follow it...

- Never (1)
- In some circumstances (2)
- In most circumstances (3)
- In all circumstances (4)

| Page Break |  |
| --- | --- |

2C As a reminder, your answers will be kept strictly confidential. If the hospital implemented a policy that **did not align** with your beliefs on allocation of ventilators, critical care beds or other limited resources, would you follow it...

- Never (1)
- In some circumstances (2)
- In most circumstances (3)
- In all circumstances (4)

| Page Break |  |
| --- | --- |

|  |
| --- |

2Da Suppose you had two patients who required ventilation, A and B, and only one ventilator.  All other factors being equal, given the following circumstances, to whom would you assign the ventilator:

|  | Definitely A (1) | Probably A (2) | Unsure (3) | Probably B (4) | Definitely B (5) |
| --- | --- | --- | --- | --- | --- |
| Patient A is a Canadian citizen / Patient B is not a Canadian citizen (1) |  |  |  |  |  |
| Patient A does not have significant comorbidities / Patient B has significant comorbidities (2) |  |  |  |  |  |
| You believe Patient A is likely to survive if ventilated / You believe Patient B is unlikely to survive if ventilated (3) |  |  |  |  |  |
| Patient A has children / Patient B does not have children (4) |  |  |  |  |  |
| Patient A is 40 with a low chance of surviving COVID / Patient B is 72 with a high chance of surviving COVID (5) |  |  |  |  |  |
| Patient A has not had a stroke and can't accomplish all activities of daily living / Patient B has had a stroke and can accomplish all activities of daily living (6) |  |  |  |  |  |

| Page Break |  |
| --- | --- |

|  |
| --- |

2Db Suppose you had two patients who required ventilation, A and B, and only one ventilator.  All other factors being equal, given the following circumstances, to whom would you assign the ventilator:

|  | Definitely A (1) | Probably A (2) | Unsure (3) | Probably B (4) | Definitely B (5) |
| --- | --- | --- | --- | --- | --- |
| Patient A has COPD / Patient B does not have COPD (1) |  |  |  |  |  |
| Patient A is cognitively intact / Patient B has dementia (2) |  |  |  |  |  |
| Patient A is married / Patient B is unmarried (3) |  |  |  |  |  |
| Patient A is a healthcare worker / Patient B is not a healthcare worker (4) |  |  |  |  |  |
| Patient A is 72 with no significant comorbidities / Patient B is 40 with COPD and heart failure (5) |  |  |  |  |  |
| Patient A does not have a history of stroke / Patient B does have a history of stroke (6) |  |  |  |  |  |

| Page Break |  |
| --- | --- |

| 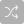 |
| --- |

2Dc Suppose you had two patients who required ventilation, A and B, and only one ventilator.  All else being equal, to whom would you assign the ventilator in each of the following cases?

|  | Definitely A (1) | Probably A (2) | Unsure (3) | Probably B (4) | Definitely B (5) |
| --- | --- | --- | --- | --- | --- |
| Patient A has heart failure / Patient B does not have heart failure (1) |  |  |  |  |  |
| Patient A is 72 / Patient B is 40 (2) |  |  |  |  |  |
| Patient A is female / Patient B is male (3) |  |  |  |  |  |
| Patient A is known to you / Patient B is unknown to you (4) |  |  |  |  |  |
| Patient A has mild heart failure / Patient B has severe heart failure (5) |  |  |  |  |  |
| Patient A can carry out all activities of daily living / Patient B can carry out some activities of daily living (6) |  |  |  |  |  |

| Page Break |  |
| --- | --- |

|  |
| --- |

2E Please rate the importance of each of the following factors for determining who receives a limited resource (e.g., critical care bed, ventilator)

|  | Not at all important (1) | Slightly important (2) | Moderately important (3) | Very important (4) | Extremely important (5) |
| --- | --- | --- | --- | --- | --- |
| Age (1) |  |  |  |  |  |
| Gender (2) |  |  |  |  |  |
| Whether patient is a Canadian citizen (3) |  |  |  |  |  |
| Whether you think patient will survive COVID-19 (5) |  |  |  |  |  |
| Whether patient has dementia (6) |  |  |  |  |  |
| Whether patient has significant comorbidities like heart failure or COPD (4) |  |  |  |  |  |
| Whether the patient can independently complete activities of daily living (13) |  |  |  |  |  |
| Whether patient is married (7) |  |  |  |  |  |
| Whether patient has children (8) |  |  |  |  |  |
| Whether patient is known to me (9) |  |  |  |  |  |
| Whether patient is a healthcare worker (11) |  |  |  |  |  |
| Other (specify) (10) |  |  |  |  |  |

| Page Break |  |
| --- | --- |

3A Are you aware of a specific protocol, policy or directive from an institution or government to guide your decision-making about who should receive limited resources (e.g., critical care beds, ventilators) in the event of a shortage?

- Yes (1)
- No (2)
- Unsure (3)

| Page Break |  |
| --- | --- |

Display This Question:

If Are you aware of a specific protocol, policy or directive from an institution or government to gu... = Yes

Or Are you aware of a specific protocol, policy or directive from an institution or government to gu... = Unsure

3B Are you aware of policies from:

|  | Yes (1) | No (2) | Unsure (3) |
| --- | --- | --- | --- |
| Your local hospital (1) |  |  |  |
| The Champlain Local Health Integration Network (LHIN) (2) |  |  |  |
| The Ontario Government (3) |  |  |  |
| The Canadian Government (4) |  |  |  |
| Global Organization (e.g., World Health Organization) (5) |  |  |  |
| Regulatory Body (e.g., College of Physicians and Surgeons of Ontario) (6) |  |  |  |
| Other (specify): (7) |  |  |  |

| Page Break |  |
| --- | --- |

3C Currently ventilators, ICU beds and personal protective equipment are being discussed as items for rationing. Which other resources do you think may need to be rationed, if any?

________________________________________________________________

| Page Break |  |
| --- | --- |

3D Please think about the most common non-pandemic-related treatments you offer in your practice. Due to pandemic conditions, how likely are you to change the way you offer any of these treatments, or stop offering them? Please select all that apply and provide specifics of treatments/conditions if you are comfortable doing so. (For example, you might enter "endovascular thrombectomy for stroke" or "surgical repair of a ruptured AAA")

- I am likely to STOP offering the following treatments: (1) ________________________________________________
- I am likely to BE MORE SELECTIVE in offering the following treatments: (2) ________________________________________________
- I am likely to CONTINUE offering the following treatments with no changes: (3) ________________________________________________
- ⊗I am unlikely to change or stop any treatments (4)

| Page Break |  |
| --- | --- |

4A In the event of resource scarcity, some patients may require palliative care if it is decided they will not receive life-sustaining therapies.
If you had to provide palliative care to someone who has been denied life-sustaining treatment due to resource scarcity, how emotionally difficult do you feel it would be?

- Not at all difficult (1)
- A little difficult (2)
- Somewhat difficult (3)
- Very difficult (4)

| Page Break |  |
| --- | --- |

4B If you had to provide palliative care to someone who has been denied life-sustaining treatment due to resource scarcity, how comfortable do you feel with each of the following?

|  | Not at all comfortable (1) | A little comfortable (2) | Somewhat comfortable (3) | Very comfortable (4) |
| --- | --- | --- | --- | --- |
| Having a goals of care conversation with the patient (8) |  |  |  |  |
| Having a goals of care conversation with the patient's family in person (9) |  |  |  |  |
| Having a goals of care conversation with the patient's family remotely (e.g. via telephone or videoconference) (10) |  |  |  |  |

| Page Break |  |
| --- | --- |

4C If you had to palliate a patient due to resource rationing who otherwise would have received a ventilator or ICU bed, how likely are you to tell the patient’s family that this decision was made due to resource scarcity?

- Not at all likely (1)
- A little likely (2)
- Somewhat likely (3)
- Very likely (4)

| Page Break |  |
| --- | --- |

5A If you had to make a decision involving allocating a health resource like ICU beds or ventilators today, who would you want to talk about it with afterward? Please select all that apply.

- Other physicians (1)
- Professional counsellor (2)
- Religious advisor (3)
- Family member (4)
- ⊗No one (5)
- Other (please specify below - multiple options possible) (6) ________________________________________________

| Page Break |  |
| --- | --- |

5B If you had to make a decision involving allocating a health resource like ICU beds or ventilators today, how confident do you feel that you could access the emotional support you required **at the time of the decision**?

- Not at all confident (1)
- A little confident (2)
- Somewhat confident (3)
- Very confident (4)

| Page Break |  |
| --- | --- |

5C If you had to make a decision involving allocating a health resource like ICU beds or ventilators today, how confident do you feel that you would be able to access the emotional support you required **after the decision**?

- Not at all confident (1)
- A little confident (2)
- Somewhat confident (3)
- Very confident (4)

| Page Break |  |
| --- | --- |

6A If you were writing a guideline on resource allocation in the time of a pandemic, what would you say is the most important thing to include?

________________________________________________________________

________________________________________________________________

________________________________________________________________

________________________________________________________________

________________________________________________________________

| Page Break |  |
| --- | --- |

7A
Finally, we would like to ask for some demographic information. You can skip any that you prefer not to answer, but please be assured that your responses will not be identifiable, as the results will be reported in aggregate form only.
Age:

- Under 35 (1)
- 35-44 (2)
- 45-54 (3)
- 55-64 (4)
- 65+ (5)
- Prefer not to answer (6)

7B Gender

- Male (1)
- Female (2)
- Nonbinary or Specify (3) ________________________________________________
- Prefer not to answer (4)

7C Field of Practice

- Medicine (2)
- Surgery (1)
- Pediatrics (3)
- Obstetrics (4)
- Psychiatry (5)
- Laboratory Medicine (6)
- Obstetrics/Gynecology (7)
- Critical Care / Anesthesia (8)
- Emergency Medicine (9)
- Family Medicine (10)
- Prefer not to answer (11)

| Page Break |  |
| --- | --- |

8A Thank you very much for your time and expertise. If you have any comments, thoughts, or anything that we have not covered, please enter them below.

________________________________________________________________

________________________________________________________________

________________________________________________________________

________________________________________________________________

________________________________________________________________

End of Block: Default Question Block
